# 4-(2-Thienylmethyl­eneamino)benzoic acid

**DOI:** 10.1107/S1600536809039208

**Published:** 2009-10-03

**Authors:** Hui Cui, Xuquan Tao

**Affiliations:** a College of Chemistry and Chemical Engineering, Liaocheng University, Shandong 252059, People’s Republic of China; b College of Materials Science and Engineering, Liaocheng University, Shandong 252059, People’s Republic of China

## Abstract

In the title mol­ecule, C_12_H_9_NO_2_S, the dihedral angle between benzene and thio­phene rings is 41.91 (8)°. The crystal packing exhibits short inter­molecular O—H⋯O and C—H⋯O hydrogen-bonding contacts.

## Related literature

For the synthesis of substituted thio­phenes, see: Koike *et al.* (1999 ▶). For the anti­cancer activity of Schiff bases, see: Chakraborty & Patel (1996 ▶). For a related structure, see: Hu *et al.* (2008 ▶).
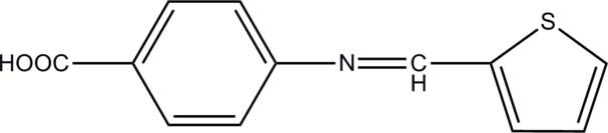


## Experimental

### 

#### Crystal data

C_12_H_9_NO_2_S
*M* *_r_* = 231.26
Monoclinic, 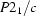

*a* = 3.8801 (3) Å
*b* = 10.0849 (11) Å
*c* = 27.380 (3) Å
β = 93.185 (1)°
*V* = 1069.74 (18) Å^3^
*Z* = 4
Mo *K*α radiation
μ = 0.28 mm^−1^
*T* = 298 K
0.43 × 0.20 × 0.12 mm

#### Data collection

Bruker SMART APEX CCD area-detector diffractometer
Absorption correction: multi-scan (*SADABS*; Sheldrick, 1996 ▶) *T*_min_ = 0.888, *T*_max_ = 0.967
5213 measured reflections
1887 independent reflections
1496 reflections with *I* > 2σ(*I*)
*R*_int_ = 0.044

#### Refinement

*R*[*F* ^2^ > 2σ(*F* ^2^)] = 0.054
*wR*(*F* ^2^) = 0.140
*S* = 1.09
1887 reflections
145 parameters
H-atom parameters constrained
Δρ_max_ = 0.35 e Å^−3^
Δρ_min_ = −0.22 e Å^−3^

### 

Data collection: *SMART* (Siemens, 1996 ▶); cell refinement: *SAINT* (Siemens, 1996 ▶); data reduction: *SAINT*; program(s) used to solve structure: *SHELXS97* (Sheldrick, 2008 ▶); program(s) used to refine structure: *SHELXL97* (Sheldrick, 2008 ▶); molecular graphics: *SHELXTL* (Sheldrick, 2008 ▶); software used to prepare material for publication: *SHELXTL*.

## Supplementary Material

Crystal structure: contains datablocks I, global. DOI: 10.1107/S1600536809039208/bq2161sup1.cif

Structure factors: contains datablocks I. DOI: 10.1107/S1600536809039208/bq2161Isup2.hkl

Additional supplementary materials:  crystallographic information; 3D view; checkCIF report

## Figures and Tables

**Table 1:** Hydrogen-bond geometry (Å, °)

*D*—H⋯*A*	*D*—H	H⋯*A*	*D*⋯*A*	*D*—H⋯*A*
O1—H1⋯O2^i^	0.82	1.83	2.641 (3)	172
C3—H3⋯O2^ii^	0.93	2.52	3.441 (4)	169

Symmetry codes: (i) 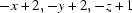; (ii) 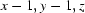.

**Fig. 1.**
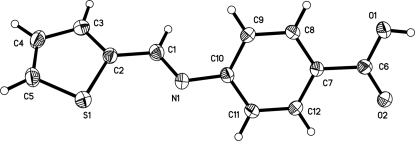
The molecular structure of (I) with the atom numbering scheme. Displacement ellipsoids are drawn at the 30% probability level.

**Fig. 2.**
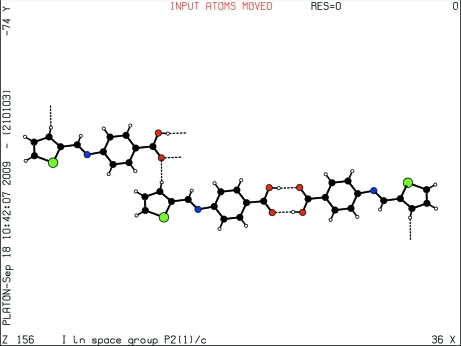
The crystal packing of (I)

## supplementary crystallographic information

### Comment

The synthesis of substituted thiophenes has attracted a great deal of interest over the years due to their presence in natural products (Koike, *et al.*, 1999). Moreover, Schiff bases derived from a large number of carbonyl compounds and amines. It has been shown that Schiff base compounds have strong anticancer activity (Chakraborty *et al.*, 1996).

Here, we report the synthesis and crystal structure of a new flexible Schiff-base compound 4-aminobenzoic acid thiophene-2-carbaldehyde schiff base, (I). The molecule of (I) is shown in Fig. 1. Bond lengths and angles are comparable with those observed in similar compounds (Hu *et al.*, 2008). The C(1)=N(1) bond length of 1.277 (4) Å, conform to the usual value for a C=N double bond. Each half of the molecule displays a *trans* configuration across the C=N double bond.

In the crystal structure, the dihedral angle between the benzene ring and the thiophene ring is 41.91 (8)°. Moreover, the two-dimensional network structures were formed by the intermolecular  O—H···O and C-H···O H-bond interactions (Figure 2 and Table 1).

### Experimental

4-Aminobenzoic acid (10 mmol), thiophene-2-carbaldehyde (10 mmol) and 20 ml ethanol were mixed in 50 ml flask. After stirring 3 h at 303 K, the resulting mixture was recrystalized from ethanol, affording the title compound as a red crystalline solid. Elemental analysis: calculated for C_12_H_9_N_2_OS: C 62.32, H 3.92, N 6.06%; found: C 62.38, H 4.14, N 6.17%.

### Refinement

All H atoms were placed in geometrically idealized positions (C—H distances are 0.93 Å, O—H distance is 0.82 Å) and treated as riding on their parent atoms, with *U*_iso_(H) = 1.2–1.5 *U*_eq_(C, O).

### Crystal data

**Table:**

C_12_H_9_NO_2_S	*F*(000) = 480
*M**_r_* = 231.26	*D*_x_ = 1.436 Mg m^−^^3^
Monoclinic, *P*2_1_/*c*	Mo *K*α radiation, λ = 0.71073 Å
Hall symbol: -P 2ybc	Cell parameters from 1697 reflections
*a* = 3.8801 (3) Å	θ = 3.0–24.6°
*b* = 10.0849 (11) Å	µ = 0.28 mm^−^^1^
*c* = 27.380 (3) Å	*T* = 298 K
β = 93.185 (1)°	Block, red
*V* = 1069.74 (18) Å^3^	0.43 × 0.20 × 0.12 mm
*Z* = 4	

### Data collection

**Table:**

Bruker SMART APEX CCD area-detector diffractometer	1887 independent reflections
Radiation source: fine-focus sealed tube	1496 reflections with *I* > 2σ(*I*)
graphite	*R*_int_ = 0.044
φ and ω scans	θ_max_ = 25.0°, θ_min_ = 1.5°
Absorption correction: multi-scan (*SADABS*; Sheldrick, 1996)	*h* = −4→4
*T*_min_ = 0.888, *T*_max_ = 0.967	*k* = −12→9
5213 measured reflections	*l* = −29→32

### Refinement

**Table:**

Refinement on *F*^2^	Primary atom site location: structure-invariant direct methods
Least-squares matrix: full	Secondary atom site location: difference Fourier map
*R*[*F*^2^ > 2σ(*F*^2^)] = 0.054	Hydrogen site location: inferred from neighbouring sites
*wR*(*F*^2^) = 0.140	H-atom parameters constrained
*S* = 1.09	*w* = 1/[σ^2^(*F*_o_^2^) + (0.0616*P*)^2^ + 0.4613*P*] where *P* = (*F*_o_^2^ + 2*F*_c_^2^)/3
1887 reflections	(Δ/σ)_max_ < 0.001
145 parameters	Δρ_max_ = 0.35 e Å^−^^3^
0 restraints	Δρ_min_ = −0.22 e Å^−^^3^

### Special details

**Table:**

Geometry. All e.s.d.'s (except the e.s.d. in the dihedral angle between two l.s. planes) are estimated using the full covariance matrix. The cell e.s.d.'s are taken into account individually in the estimation of e.s.d.'s in distances, angles and torsion angles; correlations between e.s.d.'s in cell parameters are only used when they are defined by crystal symmetry. An approximate (isotropic) treatment of cell e.s.d.'s is used for estimating e.s.d.'s involving l.s. planes.
Refinement. Refinement of *F*^2^ against ALL reflections. The weighted *R*-factor *wR* and goodness of fit *S* are based on *F*^2^, conventional *R*-factors *R* are based on *F*, with *F* set to zero for negative *F*^2^. The threshold expression of *F*^2^ > σ(*F*^2^) is used only for calculating *R*-factors(gt) *etc*. and is not relevant to the choice of reflections for refinement. *R*-factors based on *F*^2^ are statistically about twice as large as those based on *F*, and *R*- factors based on ALL data will be even larger.

### Fractional atomic coordinates and isotropic or equivalent isotropic displacement parameters (Å^2^)

**Table:**

	*x*	*y*	*z*	*U*_iso_*/*U*_eq_	
N1	0.4137 (7)	0.4083 (2)	0.34590 (9)	0.0410 (6)	
O1	0.9679 (7)	0.8245 (2)	0.50746 (8)	0.0580 (7)	
H1	1.0383	0.8943	0.5197	0.087*	
O2	0.7517 (7)	0.9632 (2)	0.45013 (7)	0.0543 (6)	
S1	0.3211 (2)	0.18720 (8)	0.26947 (3)	0.0476 (3)	
C1	0.2843 (8)	0.3008 (3)	0.36133 (11)	0.0438 (8)	
H1A	0.2297	0.2954	0.3939	0.053*	
C2	0.2192 (8)	0.1869 (3)	0.32997 (10)	0.0389 (7)	
C3	0.0828 (9)	0.0673 (3)	0.34261 (11)	0.0472 (8)	
H3	0.0114	0.0483	0.3737	0.057*	
C4	0.0618 (9)	−0.0239 (3)	0.30361 (12)	0.0506 (9)	
H4	−0.0252	−0.1094	0.3061	0.061*	
C5	0.1821 (9)	0.0267 (3)	0.26221 (12)	0.0515 (9)	
H5	0.1888	−0.0202	0.2330	0.062*	
C6	0.8065 (8)	0.8483 (3)	0.46603 (10)	0.0397 (7)	
C7	0.6845 (7)	0.7322 (3)	0.43688 (9)	0.0354 (7)	
C8	0.7347 (8)	0.6033 (3)	0.45455 (10)	0.0403 (7)	
H8	0.8320	0.5902	0.4860	0.048*	
C9	0.6413 (8)	0.4952 (3)	0.42586 (10)	0.0419 (8)	
H9	0.6774	0.4098	0.4379	0.050*	
C10	0.4922 (7)	0.5141 (3)	0.37856 (10)	0.0358 (7)	
C11	0.4398 (8)	0.6428 (3)	0.36128 (10)	0.0407 (7)	
H11	0.3413	0.6561	0.3299	0.049*	
C12	0.5320 (8)	0.7508 (3)	0.39001 (10)	0.0395 (7)	
H12	0.4924	0.8362	0.3781	0.047*	

### Atomic displacement parameters (Å^2^)

**Table:**

	*U*^11^	*U*^22^	*U*^33^	*U*^12^	*U*^13^	*U*^23^
N1	0.0479 (16)	0.0341 (14)	0.0407 (14)	−0.0028 (12)	−0.0002 (11)	−0.0039 (11)
O1	0.0861 (18)	0.0389 (13)	0.0463 (13)	−0.0045 (12)	−0.0194 (12)	−0.0047 (10)
O2	0.0839 (18)	0.0321 (12)	0.0455 (12)	−0.0027 (12)	−0.0094 (11)	−0.0006 (10)
S1	0.0579 (6)	0.0426 (5)	0.0424 (5)	−0.0005 (4)	0.0042 (4)	−0.0011 (3)
C1	0.0488 (19)	0.0427 (18)	0.0404 (16)	0.0005 (15)	0.0060 (14)	−0.0051 (14)
C2	0.0392 (17)	0.0358 (16)	0.0414 (16)	0.0019 (14)	−0.0008 (13)	−0.0027 (13)
C3	0.055 (2)	0.0433 (19)	0.0432 (17)	−0.0036 (16)	0.0038 (15)	0.0041 (14)
C4	0.055 (2)	0.0341 (17)	0.062 (2)	−0.0043 (15)	−0.0028 (17)	−0.0020 (15)
C5	0.057 (2)	0.0443 (19)	0.052 (2)	0.0033 (17)	−0.0097 (16)	−0.0126 (16)
C6	0.0469 (19)	0.0373 (17)	0.0349 (15)	0.0007 (14)	0.0025 (13)	−0.0004 (13)
C7	0.0389 (17)	0.0316 (15)	0.0356 (15)	−0.0002 (13)	0.0011 (12)	−0.0021 (12)
C8	0.0497 (19)	0.0375 (16)	0.0327 (15)	0.0014 (14)	−0.0056 (13)	0.0000 (13)
C9	0.052 (2)	0.0308 (16)	0.0426 (17)	0.0016 (14)	−0.0003 (14)	0.0016 (13)
C10	0.0376 (17)	0.0364 (16)	0.0335 (15)	−0.0015 (13)	0.0025 (12)	−0.0049 (12)
C11	0.0470 (19)	0.0400 (17)	0.0341 (15)	−0.0011 (15)	−0.0058 (13)	0.0013 (13)
C12	0.0477 (18)	0.0302 (15)	0.0403 (16)	0.0011 (14)	−0.0005 (13)	0.0020 (13)

### Geometric parameters (Å, °)

**Table:**

N1—C1	1.277 (4)	C4—H4	0.9300
N1—C10	1.414 (3)	C5—H5	0.9300
O1—C6	1.287 (3)	C6—C7	1.480 (4)
O1—H1	0.8200	C7—C12	1.396 (4)
O2—C6	1.252 (3)	C7—C8	1.397 (4)
S1—C5	1.714 (3)	C8—C9	1.380 (4)
S1—C2	1.724 (3)	C8—H8	0.9300
C1—C2	1.448 (4)	C9—C10	1.402 (4)
C1—H1A	0.9300	C9—H9	0.9300
C2—C3	1.369 (4)	C10—C11	1.392 (4)
C3—C4	1.408 (4)	C11—C12	1.379 (4)
C3—H3	0.9300	C11—H11	0.9300
C4—C5	1.350 (4)	C12—H12	0.9300
			
C1—N1—C10	120.4 (3)	O1—C6—C7	116.9 (3)
C6—O1—H1	109.5	C12—C7—C8	119.2 (3)
C5—S1—C2	91.28 (15)	C12—C7—C6	119.8 (3)
N1—C1—C2	122.4 (3)	C8—C7—C6	121.0 (2)
N1—C1—H1A	118.8	C9—C8—C7	120.7 (3)
C2—C1—H1A	118.8	C9—C8—H8	119.6
C3—C2—C1	127.3 (3)	C7—C8—H8	119.6
C3—C2—S1	110.9 (2)	C8—C9—C10	120.0 (3)
C1—C2—S1	121.8 (2)	C8—C9—H9	120.0
C2—C3—C4	113.0 (3)	C10—C9—H9	120.0
C2—C3—H3	123.5	C11—C10—C9	119.0 (3)
C4—C3—H3	123.5	C11—C10—N1	117.8 (2)
C5—C4—C3	112.4 (3)	C9—C10—N1	123.0 (3)
C5—C4—H4	123.8	C12—C11—C10	120.9 (3)
C3—C4—H4	123.8	C12—C11—H11	119.5
C4—C5—S1	112.4 (2)	C10—C11—H11	119.5
C4—C5—H5	123.8	C11—C12—C7	120.1 (3)
S1—C5—H5	123.8	C11—C12—H12	120.0
O2—C6—O1	123.0 (3)	C7—C12—H12	120.0
O2—C6—C7	120.1 (3)		
			
C10—N1—C1—C2	−176.1 (3)	O1—C6—C7—C8	1.8 (4)
N1—C1—C2—C3	179.9 (3)	C12—C7—C8—C9	1.2 (5)
N1—C1—C2—S1	1.4 (4)	C6—C7—C8—C9	−175.6 (3)
C5—S1—C2—C3	−0.4 (3)	C7—C8—C9—C10	−0.4 (5)
C5—S1—C2—C1	178.3 (3)	C8—C9—C10—C11	−0.1 (4)
C1—C2—C3—C4	−178.4 (3)	C8—C9—C10—N1	175.2 (3)
S1—C2—C3—C4	0.2 (4)	C1—N1—C10—C11	−143.8 (3)
C2—C3—C4—C5	0.2 (4)	C1—N1—C10—C9	40.8 (4)
C3—C4—C5—S1	−0.5 (4)	C9—C10—C11—C12	−0.1 (5)
C2—S1—C5—C4	0.5 (3)	N1—C10—C11—C12	−175.7 (3)
O2—C6—C7—C12	4.9 (4)	C10—C11—C12—C7	0.9 (5)
O1—C6—C7—C12	−175.0 (3)	C8—C7—C12—C11	−1.5 (5)
O2—C6—C7—C8	−178.2 (3)	C6—C7—C12—C11	175.4 (3)

### Hydrogen-bond geometry (Å, °)

**Table:**

*D*—H···*A*	*D*—H	H···*A*	*D*···*A*	*D*—H···*A*
O1—H1···O2^i^	0.82	1.83	2.641 (3)	172
C3—H3···O2^ii^	0.93	2.52	3.441 (4)	169

Symmetry codes: (i) −*x*+2, −*y*+2, −*z*+1; (ii) *x*−1, *y*−1, *z*.

## References

[bb1] Chakraborty, J. & Patel, R. N. (1996). *J. Indian Chem. Soc.* **73**, 191–195.

[bb2] Hu, S.-L., Li, Y.-T. & Cao, L.-P. (2008). *Acta Cryst.* E**64**, o115. 10.1107/S1600536807063179 PMC2915186 21200679

[bb3] Koike, K., Jia, Z., Nikaib, T., Liu, Y. & Guo, D. (1999). *Org. Lett.* **1**, 197–198.

[bb4] Sheldrick, G. M. (1996). *SADABS* University of Göttingen, Germany.

[bb5] Sheldrick, G. M. (2008). *Acta Cryst.* A**64**, 112–122. 10.1107/S0108767307043930 18156677

[bb6] Siemens (1996). *SMART* and *SAINT* Siemens Analytical X-ray Instruments Inc., Madison, Wisconsin, USA.

